# Cryptic Oral Microbiota: What Is Its Role as Obstructive Sleep Apnea-Related Periodontal Pathogens?

**DOI:** 10.3390/ijerph20031740

**Published:** 2023-01-18

**Authors:** Mayra A. Téllez Corral, Eddy Herrera Daza, Hayde K. Cuervo Jimenez, María del Mar Bravo Becerra, Jean Carlos Villamil, Patricia Hidalgo Martinez, Nelly S. Roa Molina, Liliana Otero, María E. Cortés, Claudia M. Parra Giraldo

**Affiliations:** 1 Centro de Investigaciones Odontológicas, Facultad de Odontología, Pontificia Universidad Javeriana, Bogotá D.C. 110231, Colombia; 2 Unidad de Investigación en Proteómica y Micosis Humanas, Departamento de Microbiología, Facultad de Ciencias, Pontificia Universidad Javeriana, Bogotá D.C. 110231, Colombia; 3 Faculty of Dentistry and Innovation Technology Graduate Program, Universidade Federal de Minas Gerais, Belo Horizonte 31270-901, Brazil; 4 Departamento de Matemáticas, Facultad de Ciencias, Pontificia Universidad Javeriana, Bogotá D.C. 110231, Colombia; 5 Sleep Clinic, Hospital Universitario San Ignacio and Faculty of Medicine, Pontificia Universidad Javeriana, Bogotá D.C. 110231, Colombia; 6 Departamento de Microbiología y Parasilogía, Facultad de Farmacia, Universidad Complutense de Madrid, 28040 Madrid, Spain

**Keywords:** periodontitis, obstructive sleep apnea, oral microbiota, pathogenic microbiota, chronic diseases, MALDI-TOF

## Abstract

Periodontitis has been commonly linked to periodontopathogens categorized in Socransky’s microbial complexes; however, there is a lack of knowledge regarding “other microorganisms” or “cryptic microorganisms”, which are rarely thought of as significant oral pathogens and have been neither previously categorized nor connected to illnesses in the oral cavity. This study hypothesized that these cryptic microorganisms could contribute to the modulation of oral microbiota present in health or disease (periodontitis and/or obstructive sleep apnea (OSA) patients). For this purpose, the presence and correlation among these cultivable cryptic oral microorganisms were identified, and their possible role in both conditions was determined. Data from oral samples of individuals with or without periodontitis and with or without OSA were obtained from a previous study. Demographic data, clinical oral characteristics, and genera and species of cultivable cryptic oral microorganisms identified by MALDI-TOF were recorded. The data from 75 participants were analyzed to determine the relative frequencies of cultivable cryptic microorganisms’ genera and species, and microbial clusters and correlations tests were performed. According to periodontal condition, dental-biofilm-induced gingivitis in reduced periodontium and stage III periodontitis were found to have the highest diversity of cryptic microorganism species. Based on the experimental condition, these findings showed that there are genera related to disease conditions and others related to healthy conditions, with species that could be related to different chronic diseases being highlighted as periodontitis and OSA comorbidities. The cryptic microorganisms within the oral microbiota of patients with periodontitis and OSA are present as potential pathogens, promoting the development of dysbiotic microbiota and the occurrence of chronic diseases, which have been previously proposed to be common risk factors for periodontitis and OSA. Understanding the function of possible pathogens in the oral microbiota will require more research.

## 1. Introduction

According to recent studies, patients with obstructive sleep apnea (OSA) have an increased risk of developing periodontitis [1,2]. Some hypotheses about the connection between periodontitis and OSA include a genetic predisposition, an inflammatory response that both disorders share, and a change in the oral microbiota [3].

Individuals with OSA have a higher prevalence of increased periodontal parameters such as probing depth (PD) and clinical attachment level (CAL), as well as an index of apnea–hypopnea (AHI) >15 events per hour, and they experience mouth-breathing-related oral dryness. In addition, these individuals have a microbiota characterized by an increase in Gram-negative bacteria, primarily periodontal pathogens [4,5]. It has been reported that the oral microbiota of patients with OSA is significantly different from that of individuals without OSA. The nasal microbiome of subjects with severe OSA was found to be altered and enriched with *Streptococcus*, *Prevotella*, and *Veillonella*. Several common oral commensals (*Streptococcus*, *Rothia*, *Veillonella*, and *Fusobacterium*) have been correlated with the apnea–hypopnea index [6]. Pyrosequencing has also been utilized to detect bacteria associated with OSA and hypertension, revealing the presence of *Porphyromonas* spp. and *Aggregatibacter* sp. in both mild and moderate–severe OSA [7]; both genera are associated with the development of periodontitis. Similar studies have found that patients with OSA have significantly higher levels of the *Scardovia* species [8], and that patients with OSA-comorbid hypertension have a different salivary microbiota, which includes the genera *Actinomyces*, *Absconditabacteria (SR1) [G-1]*, *Granulicatella*, *Corynebacterium*, *Peptostreptococcus*, *Porphyromonas*, and *Leptotrichia* [9].

Oral dryness affects bone remodeling triggered by hypoxia, with an increase in CO_2_ levels [5], reducing the immune system’s response to infections and allowing a higher diversity of microorganisms (bacteria and yeasts), which are capable of generating dysbiotic polymicrobial communities. Recent studies have found that individuals with OSA and periodontitis have higher levels of periodontal pathogenic bacteria [10] associated with yeasts such as *Candida* spp. [11]. Additionally, cryptic microbiota, which we describe as microorganisms that are not often considered significant oral pathogens and are not classified in the Socransky’s microbial complexes [12], nor are they associated with specific pathologies in the oral cavity, have been identified in periodontitis and OSA [11]. These cryptic microorganisms could contribute to periodontitis development in OSA patients. Therefore, the following study hypothesized about these cryptic microorganisms, which could contribute to the modulation of oral microbiota present in health or disease (periodontitis and/or OSA patients). The purpose was to analyze the presence of these cultivable cryptic oral microorganisms in individuals with periodontitis associated with OSA and to identify potential pathogens in both conditions.

## 2. Materials and Methods

### 2.1. Study Population

Demographic data, clinical oral characteristics, and cultivable cryptic microorganisms identified by MALDI-TOF equipment (Microflex from^®^ Bruker Daltonik Inc., Billerica, MA, USA) in three oral samples (saliva, subgingival plaque, and gingival sulcus) of participants from a previous study were available for re-analysis for this study. As previously described [11], participants were recruited from the Sleep Clinic of the Hospital Universitario San Ignacio and the Sleep Clinic of the Faculty of Dentistry at the Pontificia Universidad Javeriana-PUJ, Colombia. Participants with the following criteria were included: (1) adults over 30 years old, (2) individuals who have at least six teeth in their mouth, and (3) individuals who underwent a polysomnographic exam no more than six months ago. The following were the exclusion criteria: smokers, diabetics, individuals who recently took antibiotics, individuals who have had periodontal therapy in the past three months, individuals who use continuous positive airway pressure (CPAP) or bilevel positive airway pressure (BPAP), and individuals who have had pharmacological or surgical treatment for OSA. All participants were diagnosed by a sleep medicine pulmonologist and specialists in periodontics [11].

Inclusionary criteria were set to select completed clinical oral data of participants with cryptic microorganisms identified in their oral samples. Seventy-five participants that fulfilled the inclusion criteria were assigned to one of four groups according to the severity of their OSA and their periodontal diagnosis, as follows: Group 1 (G1) (H) healthy patients: non-periodontitis and non-OSA (n = 20), Group 2 (G2) (P) periodontitis and non-OSA patients (n = 13), Group 3 (G3) (OSA) OSA and non-periodontitis patients (n = 18), and Group 4 (G4) (P-OSA) periodontitis and OSA patients (n = 24).

Three oral samples (saliva, subgingival plaque, and gingival sulcus) were obtained from patients’ oral cavities as previously described [11], with no periodontal stimulation before the samples were taken (probing, prophylaxis, and calculus removal). The unstimulated saliva was collected in polypropylene tubes containing thioglycollate medium (Oxoid^®^, Thermo Fisher Scientific Inc, Waltham, MA, USA). The gingival sulcus sample was taken after the area of the tooth of interest was relatively isolated using gauze and cotton rolls. This sample was obtained by inserting standard absorbent papers (Periopapers, Oral Flow^®^, Plainview, NY, USA) into the periodontal sulcus for 30 s and vortexing them for 10 s to elute the Periopaper content into PBS before transferring them to polypropylene tubes with thioglycolate medium. Before collecting a sample of the subgingival plaque, the supragingival plaque was removed with a sterile curette and gauze. The subgingival plaque sample was then obtained with a curette and put into polypropylene tubes with thioglycolate media. The pellet from each oral sample was grown on Sabouraud agar (Merck^®^, Merck KGaA, Darmstadt, Germany) and BBL Columbia Agar^TM^ with 5% sheep blood, and incubated at 37 °C for 2 and 7 days under aerobiosis and anaerobiosis conditions, respectively. At the conclusion of the incubation period, the MALDI Biotyper^®^ system (Bruker Daltonics Inc., Billerica, MA, USA) was used to identify each type of microbial colony. The data from the 75 participants were earlier related by Corral et al, 2022 [11], and in this study, the data were analyzed in relation to cryptic oral microorganisms.

### 2.2. Data Register

The demographic data and clinical oral characteristics were recorded, including age, sex, and periodontal parameters: probing depth (PD), clinical attachment loss (CAL), plaque index (PI), bleeding of probing (BOP), and missing teeth. Additionally, the genera and species of cultivable cryptic oral microorganisms identified were recorded.

### 2.3. Statistical Analysis

The first part of the present study consisted in performing a descriptive statistic. Two-way ANOVA with Tukey’s multiple comparisons test was used to analyze the demographic data and periodontal parameters. In the second part, tests were carried out to compare the cultivable cryptic oral microorganisms between each group, determining the relative frequencies of cryptic microorganisms’ genera and species. The cluster analysis of cryptic microbial communities by the group of patients was conducted using agglomerative hierarchical clustering (AHC) according to the frequency of the microorganisms. Principal coordinates Analysis (PCoA) was calculated by the relative abundance of microorganisms. Association tests were performed within each group using the Spearman r test (*p* < 0.5) to correlate periodontal parameters and the cultivable cryptic microorganisms. The software packages GraphPad Prism 9.0.2 (GraphPad Software, San Diego, CA, USA) and XLSTAT statistical and data analysis solution (Addinsoft, New York, NY, USA) were used.

## 3. Results

### 3.1. Clinical Data

**Figure 1:**
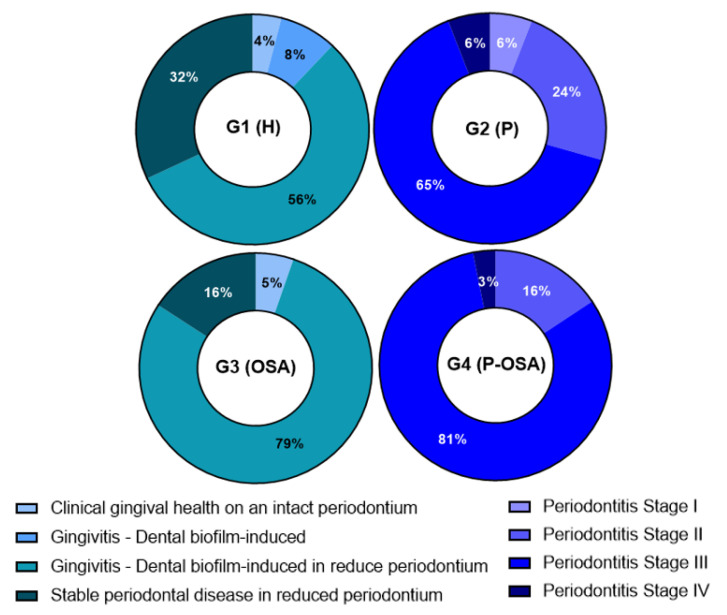
Percentage of patients of each group according to periodontal condition (according to the new Classification of Periodontal and Peri-implant Diseases and Conditions by G. Caton et al. 2018 [13]). G1 (H): healthy patients, non-periodontitis and non-OSA (n = 20); G2 (P) periodontitis and non-OSA patients (n = 13); G3: (OSA) OSA and non-periodontitis patients (n = 18); G4 (P-OSA) periodontitis and OSA patients (n = 24).

The demographic variables and periodontal parameters of the study population are presented in Table 1. There was a higher percentage of men in Group 4 (P-OSA) than in the other groups. Teeth with periodontitis (%),BOP (%), and PI showed statistically significant differences between Group 2 (P) and Group 4 (P-OSA) vs. Group 1 (H) (*p* < 0.001). In addition, the PI showed statistically significant differences between Group 3 (OSA) (*p* < 0.001) vs. Group 1 (H). Regarding the periodontal condition, dental-biofilm-induced gingivitis in reduced periodontium was more prevalent in patients in Group 1 (H) and Group 3 (OSA) (56% and 79%, respectively). Stage III periodontitis was more prevalent in patients in Group 2 (P) and Group 4 (P-OSA) (65% and 81%, respectively) (Figure 1).

### 3.2. Microbiological Data 

**Figure 2:**
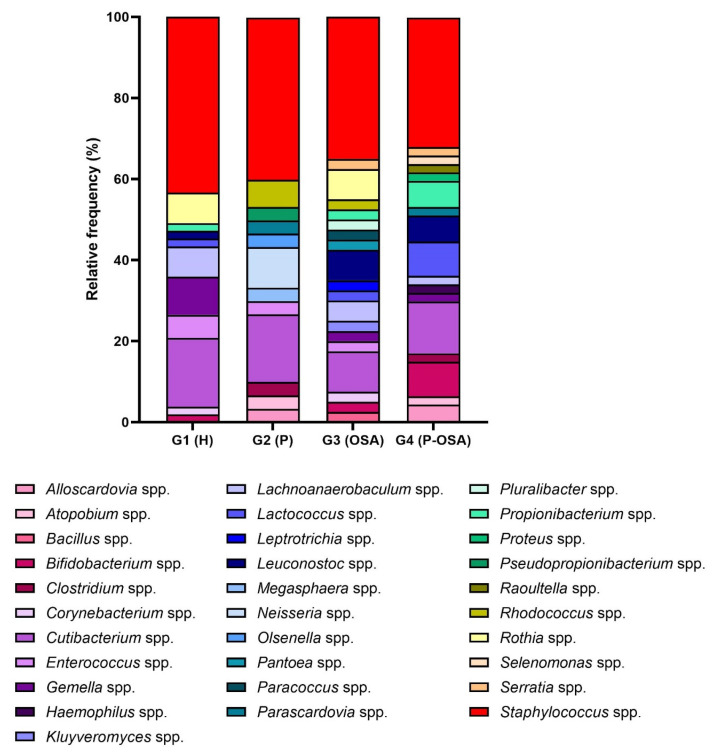
Microbial profile: percentage of each patient group’s relative frequency of the genus of cultivable cryptic microorganisms (other microorganisms). G1 (H): healthy patients; G2 (P) periodontitis patients; G3: (OSA) OSA patients; G4 (P-OSA) periodontitis and OSA patients.

According to the total number of microorganisms per genus identified by group, the percentages of the cryptic microorganisms for each group of patients were adjusted to percentages of relative frequency (Figure 2). Each patient group has a unique microbiological profile that is primarily made up of the genera *Staphylococcus* spp. and *Cutibacterium* spp.; the relative frequency of each genus ranged from 32 to 43% and 10 to 17%, respectively. The third most frequent genera in each group stood out as *Gemella* spp. (9%) in G1 (H), *Neisseria* spp. (10%) in G2 (P), *Rothia* spp., *Leuconostoc* spp. (7.5%) in G3 (OSA), and *Bifidobacterium* spp. and *Lactococcus* spp. (8.5%) in G4 (P-OSA). There was a decrease in the presence of four genera in G2 (P), G3 (OSA), and G4 (P-OSA) compared to G1 (H): *Staphylococcus* spp.: 3% decrease in G2 (P), 8.4% in G3 (OSA) and 11.5% in G4 (P-OSA); *Enterococcus* spp.: 2.3% decrease in G2 (P), 3.2% in G3 (OSA) and 5.7% in G4 (P-OSA); *Gemella* spp.: 9.4% decrease in G2 (P), 6.9% in G3 (OSA) and 7.3% in G4 (P-OSA); *Lachnoanaerobaculum* spp.: 7.6% decrease in G2 (P), 2.6% in G3 (OSA) and 5.4% in G4 (P-OSA). Otherwise, there was an increase in the presence of five genera in G2 (P), G3 (OSA), and G4 (P-OSA) compared to G1 (H): *Alloscardovia* spp.: 3.3% increase in G2 (P) and 4.3% in G4 (P-OSA); *Bifidobacterium* spp.: 0.6% increase in G3 (OSA) and 6.6% in G4 (P-OSA); *Corynebacterium* spp.: 0.6% increase in G3 (OSA); *Lactococcus* spp.: 0.6% increase in G3 (OSA) and 6.6% in G4 (P-OSA); *Leuconostoc* spp.: 5.6% increase in G3 (OSA) and 4.5% in G4 (P-OSA); *Propionibacterium* spp.: 0.6% increase in G3 (OSA) and 4.5% in G4 (P-OSA). 

**Figure 3:**
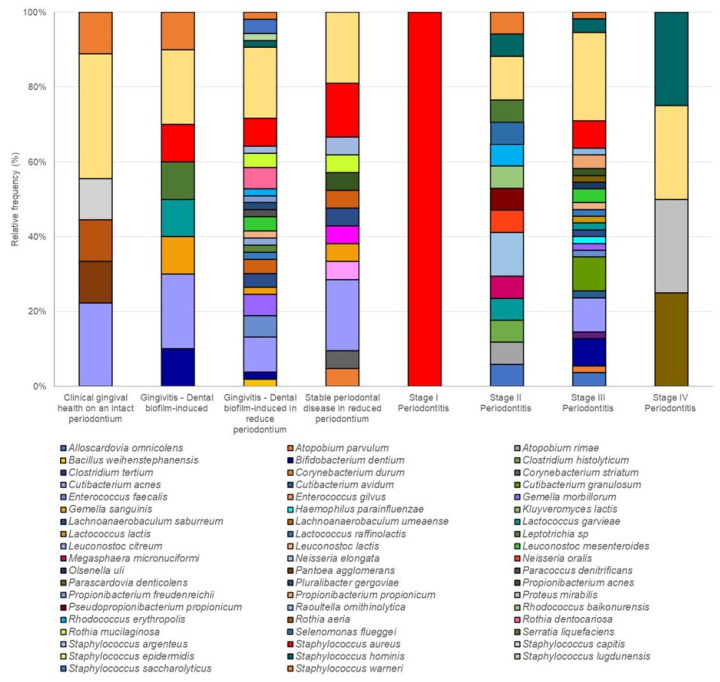
Microbial profile: percentage of relative frequency of each species of cultivable oral cryptic microorganisms by periodontal condition. Healthy conditions: clinical gingival health on an intact periodontium, dental-biofilm-induced gingivitis, dental-biofilm-induced gingivitis in reduced periodontium, Stable periodontal disease in reduced periodontium. Disease condition: periodontitis Stages I–IV.

Some species were only identified in one of the four patient groups: (Table S1). Fifty-six species were found, distributed into thirty-one genera. The major diversity of species of cryptic microorganisms was identified in dental-biofilm-induced gingivitis in reduced periodontium (26 species) and in stage III periodontitis (25 species), with *Staphylococcus epidermidis*, *Cutibacterium acnes*, and *Bifidobacterium dentium* being the most prevalent in both conditions (Figure 3).

**Figure 4:**
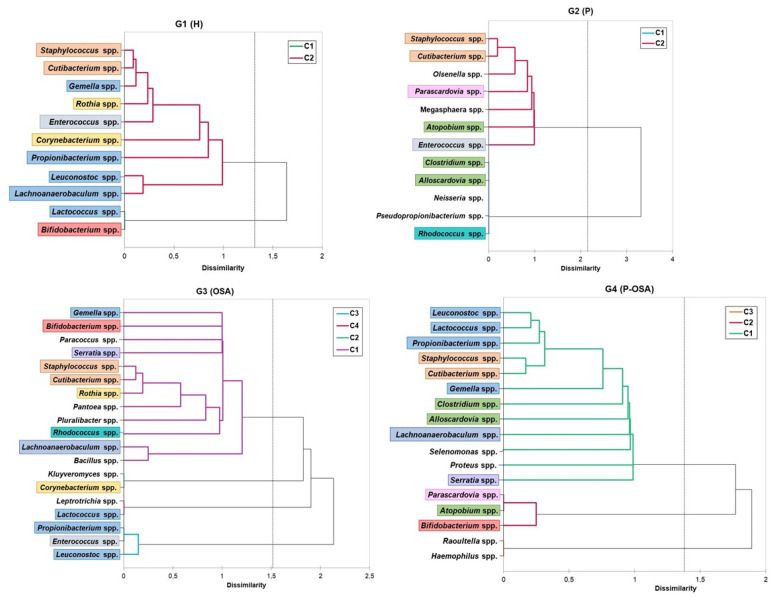
Microbial profiles dendrogram: clustering of oral cryptic microorganisms based on Euclidean distance dissimilarity matrix and agglomeration method of Ward (agglomerative hierarchical clustering (AHC)). The microorganisms present in two or more patient groups are indicated by color. Those without color were only identified in a group. C: cluster; G1 (H): healthy patients; G2 (P) periodontitis patients; G3: (OSA) OSA patients; G4 (P-OSA) periodontitis and OSA patients.

The relative frequencies of microorganisms were analyzed using agglomerative hierarchical clustering (AHC) in order to classify cryptic microorganisms based on the microbial community makeup by patient group. The results of this study are depicted as a dendrogram (Figure 4), which illustrates the degree to which the community makeup differs and how the cryptic microorganisms clustered for each patient group. The ensuing G1 (H) and G2 (P) dendrograms revealed two major well-defined clusters. In Cluster 2, *Staphylococcus* spp., *Cutibacterium* spp., and *Enterococcus* spp. Were shared by both groups. G3 (OSA) displayed four clusters, while G4 (P-OSA) displayed three clusters, with *Bifidobacterium* spp. Constituting Cluster 1 in G3 (OSA) and Cluster 2 in G4 (P-OSA).

In order to observe the distributions of the cryptic microbiota in each category, PcoA was applied to the relative abundances in each group of patients (Figures S1–S4). The G1 scattering showed that Component 1 was defined by *Bifidobacterium* spp., *Lactococcus* spp. and *Gemella* spp., and *Enterococcus* spp., and Component 2 was defined by *Corynebacterium* spp. However, when applying marimax rotation, this component was redefined *by Lachnoanaerobaculum* spp. and *Leuconostoc* spp., the distribution for men and women being associated with Component 1. The G2 scattering showed that *Alloscardovia* spp., *Clostridium* spp., *Neisseria* spp., and *Pseudopropionibacterium* spp. Highly defined the first component, and the component was mainly defined by *Staphylococcus* spp. This was maintained in rotation, being directly associated with those of stage II periodontitis. The G3 scattering showed that Component 1 was defined by *Enterococcus* spp, *Leuconostoc* spp., *and Propionibacterium* spp., being highly associated with moderate OSA and inversely with severe OSA and women. Component 2 was mainly defined by *Staphylococcus* spp., and this is maintained in rotation, being inversely associated with men. The G4 scattering showed that *Haemophilus* spp., *Staphylococcus* spp., and *Raoultella* spp. Defined Component 2 with an associative pattern for men and women, while *Bifidobacterium* spp., *Atopobium* spp., and *Parascardovia* spp. Defined Component 1 with an association trend with severe OSA.

### 3.3. Association between Periodontal Parameters and the Cryptic Oral Microorganisms

**Figure 5:**
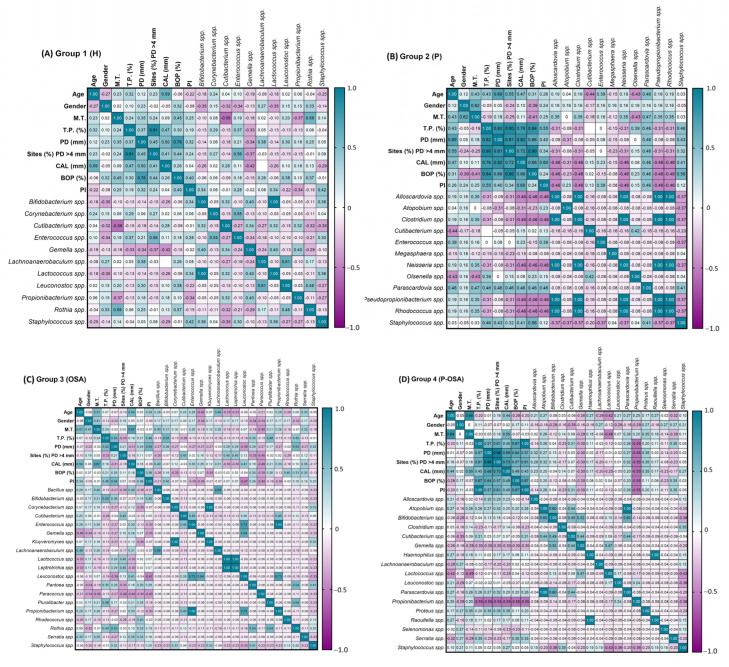
Multicomponent matrix for the correlation of periodontal parameters and oral cryptic microorganisms present in each of the groups of patients evaluated: (**A**) G1 (H), n = 20; (**B**) G2 (P), n = 13; (**C**) G3 (OSA), n = 18; (**D**) G4 (P-OSA), n = 24, using the Spearman’s rank correlation coefficient r_s_ > 0.30, *p* < 0.05. The association can be positive (+) or negative (−) according to the Spearman correlation range (r_s_). The r_s_ values over zero indicate a positive correlation in cyan tones, whereas rs values below zero indicate a negative correlation in purple tones. M.T., Missing teeth; T.P., Teeth with periodontitis; PD: probing depth; CAL: clinical attachment loss; BOP: bleeding of probing; PI: plaque index.

This correlation between periodontal parameters and the genera of species of cryptic microorganisms present in the four groups of patients evaluated was found by the analysis of multicomponent matrices. The association can be positive (+) or negative (−) according to the Spearman correlation range (r_s_). The rs values over zero indicate a positive correlation in cyan tones, whereas r_s_ values below zero indicate a negative correlation in purple tones. (Figure 5, Table S2).

In G1 (H), there was a positive, statistically significant correlation between missing teeth and *Rothia* spp. (r_s_ = 0.58, *p* = 0.004), PD (mm), and *Lachnoanaerobaculum* spp. (r_s_ =0.38, *p* = 0.047), sites (%) PD > 4 mm and *Enterococcus* spp. (r_s_ = 0.56, *p* = 0.006), BOP (%) and *Leuconostoc* spp. (r_s_ = 0.38, *p* = 0.048), and PI and *Staphylococcus* spp. (r_s_ = 0.42, *p* = 0.032), whereas *Cutibacterium* spp. And missing teeth, and CAL and *Gemella* spp. Showed a negative statistically significant correlation (r_s_ = −0.56, *p* = 0.005 and r_s_ = −0.42, *p* = 0.032, respectively) (Figure 5A). In G2 (P), there was a positive correlation with statistical significance between *Staphylococcus* spp. And teeth with periodontitis (%) (r_s_ = 0.46, *p* = 0.056), as with BOP (%) (r_s_ = 0.56, *p* = 0.026) (Figure 5B). In G3 (OSA), there was a positive correlation with statistical significance between age and *Lachnoanaerobaculum* spp. (r_s_ = 0.49, *p* = 0.018), missing teeth and *Bacillus* spp. (r_s_ = 0.40, *p* = 0.05), teeth with periodontitis (%), and *Bifidobacterium* spp. (r_s_ = 0.55, *p* = 0.01), as with *Pluralibacter* spp. and *Rothia* spp. (r_s_ = 0.48, *p* = 0.022; r_s_ = 0.43, *p* = 0.036, respectively). In addition, PD (mm) and *Rothia* spp. (r_s_ = 0.36, *p* = 0.011), sites (%) PD > 4 mm, and *Cutibacterium* spp. (r_s_ = 0.45, *p* = 0.03), whereas there was a negative statistical significance between *Leuconostoc* spp. And PD (mm) (r_s_ = −0.39, *p* = 0.046), as with PI (r_s_ = −0.47, *p* = 0.023) (Figure 5C). In G4 (P-OSA), *Proteus* spp. Was correlated positively with missing teeth, PD (mm) and sites (%) PD > 4 mm (r_s_ = 0.56, *p* = 0.026). *Serratia* spp. Was correlated positively with teeth with periodontitis (%), BOP (%) and PI (r_s_ = 0.35, *p* = 0.048), whereas *Lactococcus* spp. Was correlated negatively with missing teeth (r_s_ = −0.49, *p* = 0.008) and *Propionibacterium* spp. With all the periodontal parameters (r_s_ = −0.55, *p* = 0.003) (Figure 5D).

## 4. Discussion

This study is the first to analyze the presence of microorganisms found in oral samples that are not often linked to oral diseases such as periodontitis related to OSA. A previous study [11] demonstrated that there was a greater diversity of microorganisms in oral samples from individuals with periodontitis and OSA, and showed the association between both diseases by sharing risk factors such as comorbidities and presence of the bacteria of the orange and red complexes, associated with *Candida albicans*. Additionally, this study identified cultivable cryptic microorganisms in healthy individuals (G1), individuals with periodontitis (G2), individuals with OSA (G3), and individuals with periodontitis and with OSA (G4), which describe as microorganisms that are not often considered significant oral pathogens and are not classified in Socransky’s microbial complexes [12], nor are they associated with specific pathologies in the oral cavity; however, they could be microorganisms that contribute to periodontitis development in OSA patients and could be associated with other chronic pathologies. It is crucial to clarify the role and modulation of this diverse group of microorganisms [14,15,16,17,18,19,20,21], which goes beyond those first identified as periodontal pathogens, in both disease and pathogenicity, taking into consideration the effectiveness of current identification technologies. It is important to consider that cryptic microorganisms may or may not contribute to the health and emergence of periodontal disease or may not. To clarify the role of these microorganisms, the purpose of the current study was to analyze their presence in health or disease (periodontitis and/or OSA) and to identify potential pathogens.

The periodontal parameters BOP (%) and PI were highest in all groups of patients, compared to patients of G1, highlighting that the patients of G3 had an increased PI with a statistically significant difference (*p* = 0.001). This evidence suggests that OSA might favor oral biofilm formation, and is consistent with previous reports [22,23]. In addition, other study demonstrated that periodontal parameters such as PD (mm) and CAL were higher in patients with OSA [4], supporting the idea that OSA pathophysiology, which includes hypoxia, hypercapnia, and oral dryness, can contribute to the development of periodontitis.

According to the new Classification of Periodontal and Peri-implant Diseases and Conditions [13], the periodontal condition of the patients of each group was determined. The dental-biofilm-induced gingivitis in reduced periodontium was more frequent in G1 and G3 (56% and 79%, respectively). Meanwhile, stage III periodontitis was more frequent in G2 and G4 (65% and 85%, respectively). These results suggest that OSA is a factor that increases the risk of periodontitis, and it is crucial to comprehend that OSA individuals should undergo periodic periodontal screenings. The percentage of the relative frequency of each species of cultivable oral cryptic microorganisms showed that dental-biofilm-induced gingivitis in reduced periodontium as a healthy condition and stage III periodontitis as a disease condition had major diversity of species.

There was evidence that the presence and absence of certain species vary depending on health and disease conditions, as a possible modulation between the cryptic microorganisms. According to the literature, the severity of OSA may be involved in the diversity and abundance of different species and genera of microorganisms [5]. This could be related to hypoxic episodes and an increase in CO_2_ levels, which are consequences of apnea or hypopnea events, reflected in AHI, which determines the severity of OSA. The oral samples analyzed in a previous study [11] were taken at the same time in the morning to minimize potential influences on the microbiota’s characterization and take into consideration the possibility of diurnal oscillations in their composition and function [24]. The experimental data obtained are reproducible once this factor is taken into consideration along with the sample collection techniques.

Different studies have reported alterations in oral microbiota evaluating just the saliva of patients with OSA. Nizam et al. reported the results of 13 patients without OSA, 17 patients with mild–moderate OSA, and 22 patients with severe OSA [5]. Ko et al. concluded oral microbiota changes from the saliva of 19 patients with OSA [25], and Chen et al. found alterations in the salivary microbiome in 26 patients with OSA comorbid hypertension [9]. In the current study, the oral microbiota data were complemented, analyzing three oral samples (saliva, subgingival plaque, and gingival sulcus) from 75 patients categorized into four groups based on the diagnosis of periodontitis and OSA: 20 healthy patients (G1), 13 patients with periodontitis (G2), 18 patients with OSA (G3), and 24 patients with periodontitis and OSA (G4). Therefore, the cryptic microorganisms identified were analyzed, according to the presence of each bacterium and its association with healthy or disease conditions, to identify potential pathogens, which may be related to the presence of periodontitis associated with OSA.

According to the results of the cluster conformation, periodontitis and OSA affect the diversity and distribution of cryptic microorganisms and unique genera in each group formed the majority of clusters. In G2 (P), both genera *Olsenella* spp. And *Megasphaera* spp. have been reported as periodontal pathogens. *M. micronuciformis* has been isolated from women suffering from preterm birth [26,27]. In G3 (OSA), the microorganisms *Paracoccus* spp., *Pantoea* spp., *Pluralibacter* spp., and *Bacillus* spp. are related to biofilm formation, abscess, bacteremia, pneumonia, urinary tract infection, septic arthritis, osteomyelitis, peritonitis, choledocholithiasis, dacryocystitis, and endophthalmitis [28]. *Bacillus* sp. Have pathogenic potential through the production of enterotoxins [15,29,30]. In G4 (P-OSA), *Selenomonas* spp. And *Proteus* spp. are related to pathogenesis of periodontal disease [31] and catheter-associated urinary tract infections (CAUTIs) [32], respectively. Furthermore, other microorganisms were found in each group, but in small clusters. In G2, the genus *Neisseria* spp. has been related to endocarditis and osteomyelitis [33], and *N. oralis* has been identified in systemic infection and cystitis in a diabetic adult [34]. *Leptotrichia* sp. (G3) has been related to periodontal disease and abscesses of the oral cavity, endocarditis, and septicemia [35], and *Kluyveromyces* sp. (G3) has been identified as a yeast producer of pGK1 killer toxin [36]. *Raoultella* spp. (G4) has been related to infected root canals and urinary, gastrointestinal, hepatobiliary, and osteoarticular infections [37], and *Haemophilus* spp. (G4) has been related to endocarditis, meningitis, pneumonia, otitis media, sinusitis, and epiglottitis [38]. The PcoA identified that *Alloscardovia* spp., *Clostridium* spp., *Neisseria* spp., and *Staphylococcus* spp. have been associated with stage III periodontitis in G2 (P). *Alloscardovia* spp. Has been related to dental caries [14], and *Clostridium* spp. has been related to gas gangrene, bacteremia, meningitis, septic arthritis, enterocolitis, spontaneous bacterial peritonitis, post-traumatic brain abscess, and pneumonia [16,17]. *Enterococcus* spp., *Leuconostoc* spp., and *Propionibacterium* spp. have been associated with moderate OSA in G3 (OSA). *Enterococcus* spp. has been related to endodontic disease, bacteremia, endocarditis, urinary tract infections, diabetic foot ulcers, and cholecystitis [20,21], and *Leuconostoc* spp. has been related to bacterial meningitis and bacteremia [39,40,41]. These results suggest that severe conditions of periodontitis and OSA may harbor microorganisms that favor the development of systemic infectious disease.

Regarding the cryptic microorganisms identified (Table S3) [18,19,42,43,44,45,46,47,48], *Staphylococcus* spp. And *Cutibacterium* spp. Were the genera more common in all patient groups. However, it was clear that their abundance was higher in G1 compared to the other patients’ groups. *Staphylococcus* spp. Is frequent in skin and soft tissue infections, bloodstream infections, endocarditis, osteomyelitis, lung infection, suppurative diseases, pneumonia, prosthetic joint infections, and toxic shock syndrome [49,50,51,52,53,54,55,56,57]. *S. aureus* stands out in this genus, considered an opportunistic pathogen that is a part of the skin and nasal microbiota. It has been related to infectious diseases of the oral cavity, such as periodontitis [26], coinciding with the positive correlation found between *Staphylococcus* spp. And TP (%) and with BOP (%) in G2, and an in vitro study determined that *S. aureus* has the ability to bind to periodontal pathogenic bacteria, such as *F. nucleatum* and *P. gingivalis*, supporting the idea that *S. aureus* can become part of the complex oral microbiota and contribute to the development of oral infections [58].

The presence of *S. epidermidis* and *S. hominis* is associated with skin conditions such as atopic dermatitis or psoriasis; bloodstream infections, including endocarditis, peritonitis, and osteomyelitis; and infections of the bones and joints [52,53]. Both bacteria have been isolated from subgingival samples of healthy and periodontitis patients, without significant differences between both conditions [59], also supported by our results. The current study found that patients with periodontitis and OSA had lower levels of *Staphylococcus* spp. A highly diverse microbial community that influences the microenvironment and controls this bacterium’s growth may be responsible for this bacterium’s decline. Furthermore, a study found that *Staphylococcus* spp. Increased during treatment with continuous positive airway pressure (CPAP), the primary therapy for OSA patients [25]. This finding might explain the increase of *Staphylococcus* spp. In healthy patients due to the restored microenvironment that favors its growth.

*Gemella* spp. Is mainly linked to poor dental health, dental manipulation or surgery, colorectal disease or procedures, steroid therapy, diabetes mellitus, or hepatocellular dysfunction [60,61]. Moreover, *Gemella* spp. Is also linked to septic arthritis and oral abscesses, which can result in serious endovascular infections such as endocarditis and pericarditis [62]. CPAP-using OSA patients were shown to have lower levels of this bacterium [25]. In contrast, the healthy patients (G1) in the current study had a higher prevalence of these bacteria than in the other patient groups. Like this, a study found a higher proportion of *Gemella* sp. In saliva from healthy patients than in the saliva obtained from periodontitis patients. This study demonstrated that *Gemella* sp. in saliva is linked to periodontal health and the protein components in *Gemella*’s culture supernatant directly inhibited *P. gingivalis*’s growth in vitro [63].

In the current study, *Cutibacterium* spp. and *Propionibacterium* spp. [64] were independently identified by MALDI-TOF. Infections in neurosurgical shunts, bone, breast, and prostate infections; infective endocarditis; splenic and cutaneous abscesses; and chronic blepharitis and endophthalmitis are all linked to *Cutibacterium* spp. [65,66,67]. *C. acnes* plays a role in the onset and development of Alzheimer’s disease (AD) [68] and Parkinson’s disease (PD) [69], and this bacterium has been demonstrated to be able to cross the blood–brain barrier through transcellular invasion in an in vitro study [70]. In addition, *C. acnes* is recognized for its ability to form biofilms on biomaterials in implanted medical devices, such as *C. albicans* [71]. These two microorganisms can establish polymicrobial biofilms that are synergistic, which enhances yeast resistance to micafungin [72]. The frequency of *C. albicans* and the percentage of biofilm were both higher in patients with periodontitis and OSA [11]; in this scenario, *C. acnes* could participate as a *C. albicans* protector, encouraging the development of dysbiotic biofilms. This might lend to the notion that these opportunistic microorganisms behave in a way that makes it possible for periodontopathogen bacteria to colonize in periodontitis linked to OSA.

*Propionibacterium* spp. live on human skin as well as in the gastrointestinal and oral mucosa [73,74]. They have been linked to endocarditis and the infection of both natural and artificial valves [75], and are present in endodontic infections with a higher prevalence for secondary endodontic lesions [76]. In the current study, *Propionibacterium* spp. was identified in patients with OSA and patients with periodontitis and OSA, increasing by 0.61% in G3 and 4.5% in G4. The presence of this microorganism in individuals with OSA and those who also had periodontitis and OSA is reported for the first time in this study. Another study determined the presence of *Propionibacterium* spp. in apical periodontitis, forming a network of interactions with *Lactobacillus* spp. and different species of *Streptococcus* [77]. It is crucial to emphasize the link between the presence of this microorganism in co-association with other periodontal pathogens in periodontitis and OSA. According to reports [78,79], OSA is associated with chronic diseases such as AD and PD, primarily due to its potential neurodegenerative effects. In a similar vein, studies have suggested that periodontitis and AD may have a potential bidirectional relationship that may be caused by microorganisms [80]. The present study opens the possibility of establishing microbiological implications; for instance, the presence of *P. acnes* may play a role in the development and progression of this type of neurological disease in OSA patients.

*Bifidobacterium* spp. is usually related to dental caries, by acidogenic potential [81,82], and is a well-adapted commensal in the gastrointestinal tract. An earlier study determined that *Bifidobacterium* dentium is associated with an increased risk for gastrointestinal cancer [83], and along with *Lactobacillus*, *Bacteroides*, and *Prevotella*, was detected in OSA patients. It has also been implicated in obesity and diabetes [84]. Moreover, a substantial correlation between *B. dentium* abundance and pregnant women’s salivary progesterone concentrations [85] and also pregnant women who had *B. dentium* had greater levels of IL-6 and IL-8 [86]. G3 and G4 had the highest frequency of *Bifidobacterium* sp. compared to G1 and G2, and it was positively correlated with teeth with periodontitis (%) and PI in G3 and the presence of *Cutibacterium* spp. in G4, indicating its pathogenic effect in both diseases, and microorganisms with *Atopobium* spp. [87,88] and *Parascardovia* spp. [89] showed an association trend with severe OSA.

Despite these findings, more research will be required to fully understand the connections between cryptic oral microorganisms and known oral pathogens in periodontitis associated with OSA, as well as their participation as risk factors.

## 5. Conclusions

This study reveals the existence of cryptic microorganisms and confirms that some of them are connected to a microbial profile of health, while others are more connected to a microbial profile of sickness. While *Gemella* spp. were connected to healthy conditions, *Cutibacteria* spp., *Propionibacterium* spp., and *Bifidobacterium* spp. were connected to disease profiles. *Staphylococcus* spp. prevalence in all patient categories may vary depending on the presence of other microorganisms. It was discovered that the illness profile contained microorganisms that might be categorized as possibly pathogenic and may have a role in the interaction between OSA, periodontitis, and other clinically significant chronic disorders or systemic infectious diseases. Understanding whether and how they will respond to health and disease is based on this reality.

## Figures and Tables

**Table 1:** Demographic variables and periodontal parameters of the group of patients.

Clinical Variable	Group 1 (H) (n = 20)	Group 2 (P) (n = 13)	Group 3 (OSA) (n = 18)	Group 4 (P-OSA) (n = 24)
Age (years)	44.35 ± 14.24	40.69 ± 10.83	50.35 ± 13.09	49.33 ± 11.65
Gender (Males) (%)	32	41	37	69
Missing teeth	6.21 ± 4.42	5.69 ± 2.25	8.53 ± 6.53	7.08 ± 6.23
Teeth with periodontitis (%)	2.18 ± 3.99	47.91 ± 24.72 *	1.37 ± 2.98 ^»^	39.16 ± 19.39 *^,§^
PD (mm)	1.81 ± 0.48	2.64 ± 0.45	2.36 ± 4.82	13.83 ± 9.95
Sites (%) PD ≥ 4 mm	0.29 ± 0.50	16.76 ± 10.62	2.01 ± 0.15	2.64 ± 0.41
CAL (mm)	1.33 ± 0.78	2.15 ± 1.01	1.52 ± 0.98	2.31 ± 1.14
BOP (%)	11.59 ± 11.46	49.19 ± 26.92 *	24.02 ± 22.96 ^»^	48.67 ± 26.69 *^,§^
PI	19.30 ± 11.48	47.21 ± 26.28 *	39.65 ± 21.43 *	39.16 ± 19.39 *

Values are given as mean ± standard deviation; Two-way ANOVA, Tukey’s multiple comparisons test, *p* < 0.05; PD: Probing depth; CAL: Clinical attachment loss; BOP: Bleeding of probing; PI: Plaque index; * Significantly different to Group 1; ^»^ Significantly different to Group 2; ^§^ Significantly different to Group 3.

## Data Availability

Not applicable.

## Supplementary Materials

The following supporting information can be downloaded at: https://www.mdpi.com/article/10.3390/ijerph20031740/s1, Figure S1: Principal coordinates analysis (PCoA) calculated by the relative abundance of microorganisms in G1 (H). Figure S2: Principal coordinates analysis (PCoA) calculated by the relative abundance of microorganisms in G2 (P). Figure S3: Principal coordinates analysis (PCoA) calculated by the relative abundance of microorganisms in G3 (OSA). Figure S4: Principal coordinates analysis (PCoA) calculated by the relative abundance of microorganisms in G4 (P-OSA). Table S1: Species identified in each group of patients. Table S2: Positive and negative correlations between the genera of microorganisms and periodontal parameters in each group of patients. Table S3: Identification of cryptic microorganism species and their relation to disease.
