# Supplementary material for: Cryptic Oral Microbiota: What Is Its Role as Obstructive Sleep Apnea-Related Periodontal Pathogens?
Source: Int J Environ Res Public Health. 2023 Jan 18;20(3):1740. doi: 10.3390/ijerph20031740 (PMC9913967; doi:10.3390/ijerph20031740)
Supplement: Supplementary file 1 [file ijerph-20-01740-s001.zip › ijerph-2089346-supplementary.pdf]

Cryptic oral microbiota: What is its role as OSA-related periodontal pathogens?

Mayra A. Téllez Corral, Eddy Herrera Daza, Hayde K. Cuervo Jimenez, María del Mar Bravo, Jean Carlos Villamil, Patricia Hidalgo Martinez, Nelly S. Roa Molina, Liliana Otero, Maria E. Cortés, Claudia M. Parra Giraldo

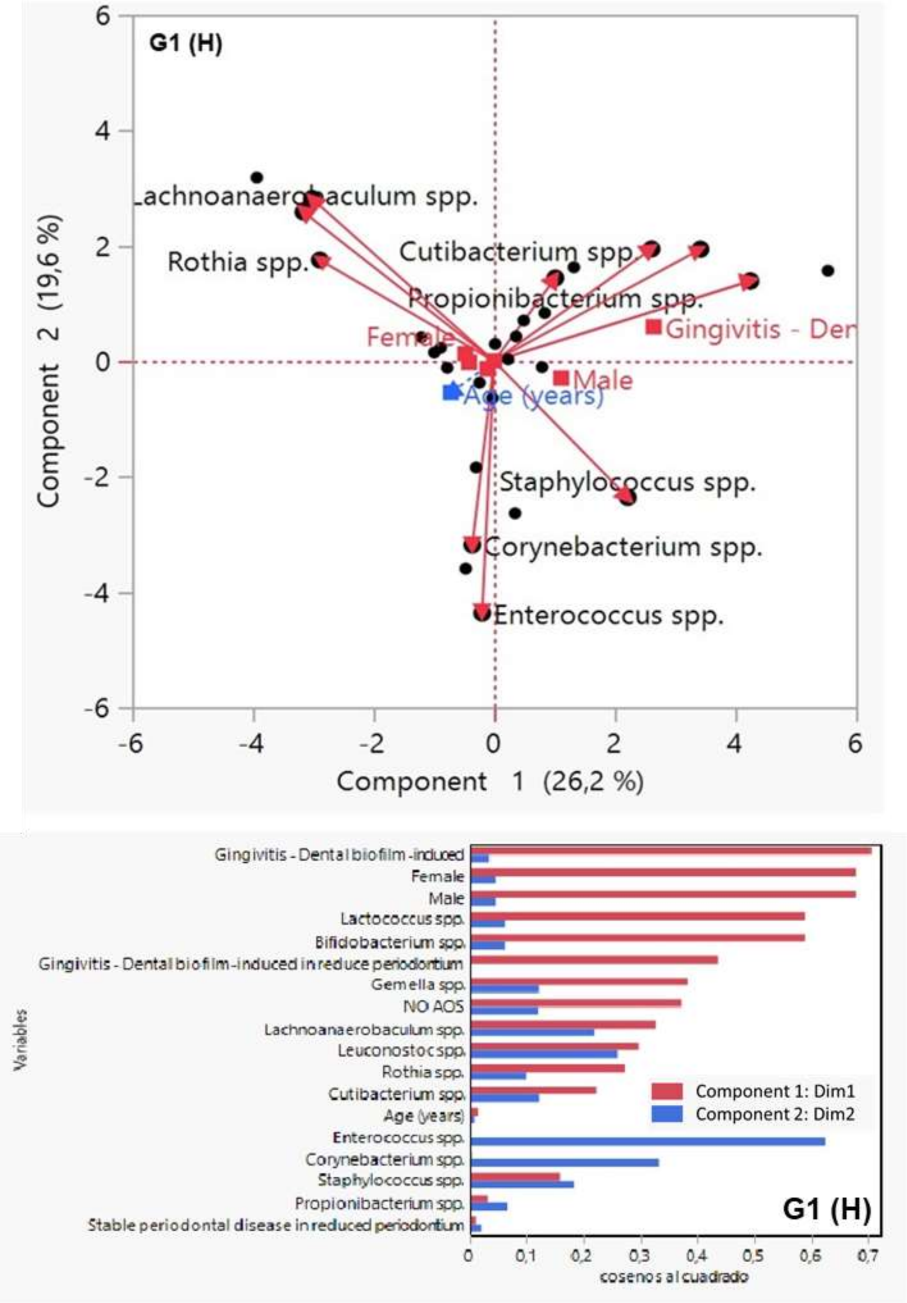

Figure S1. Principal Coordinates Analysis (PCoA) calculated by the relative abundance of microorganisms in G1 (H).

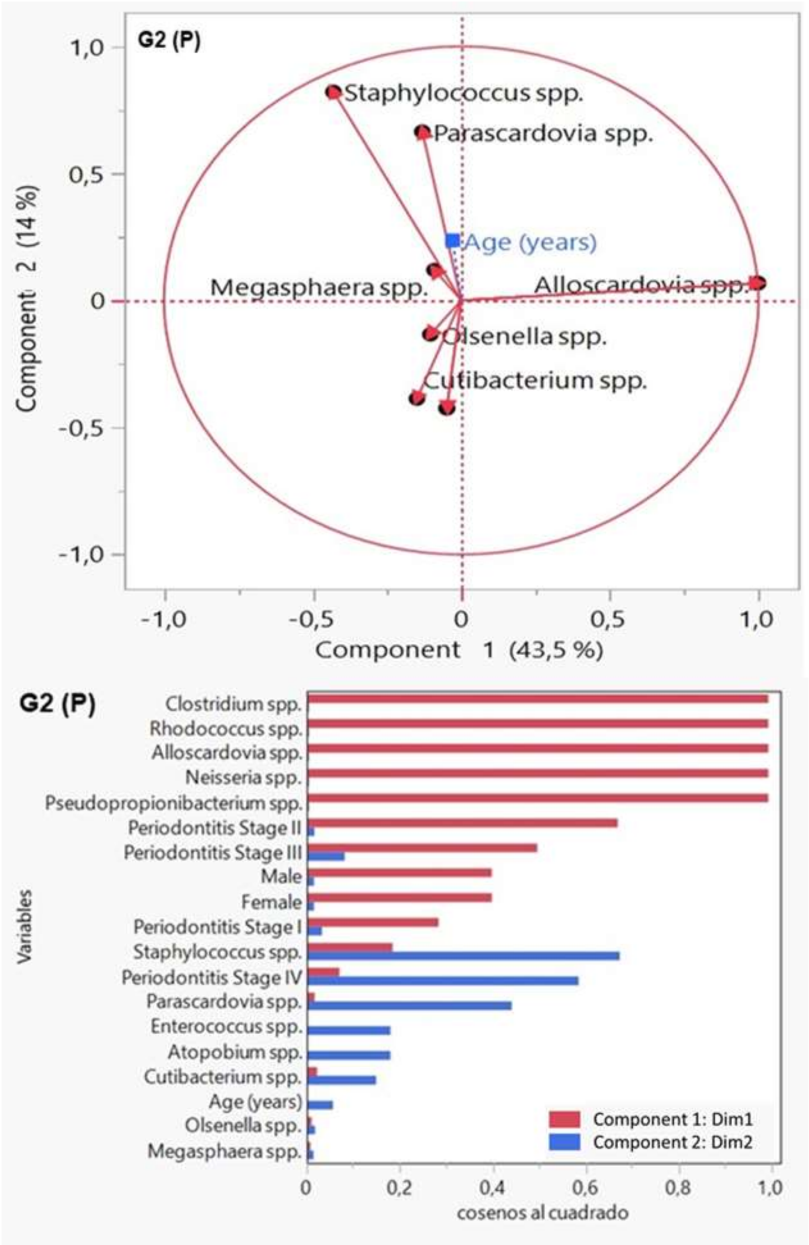

Figure S2. Principal Coordinates Analysis (PCoA) calculated by the relative abundance of microorganisms in G2 (P).

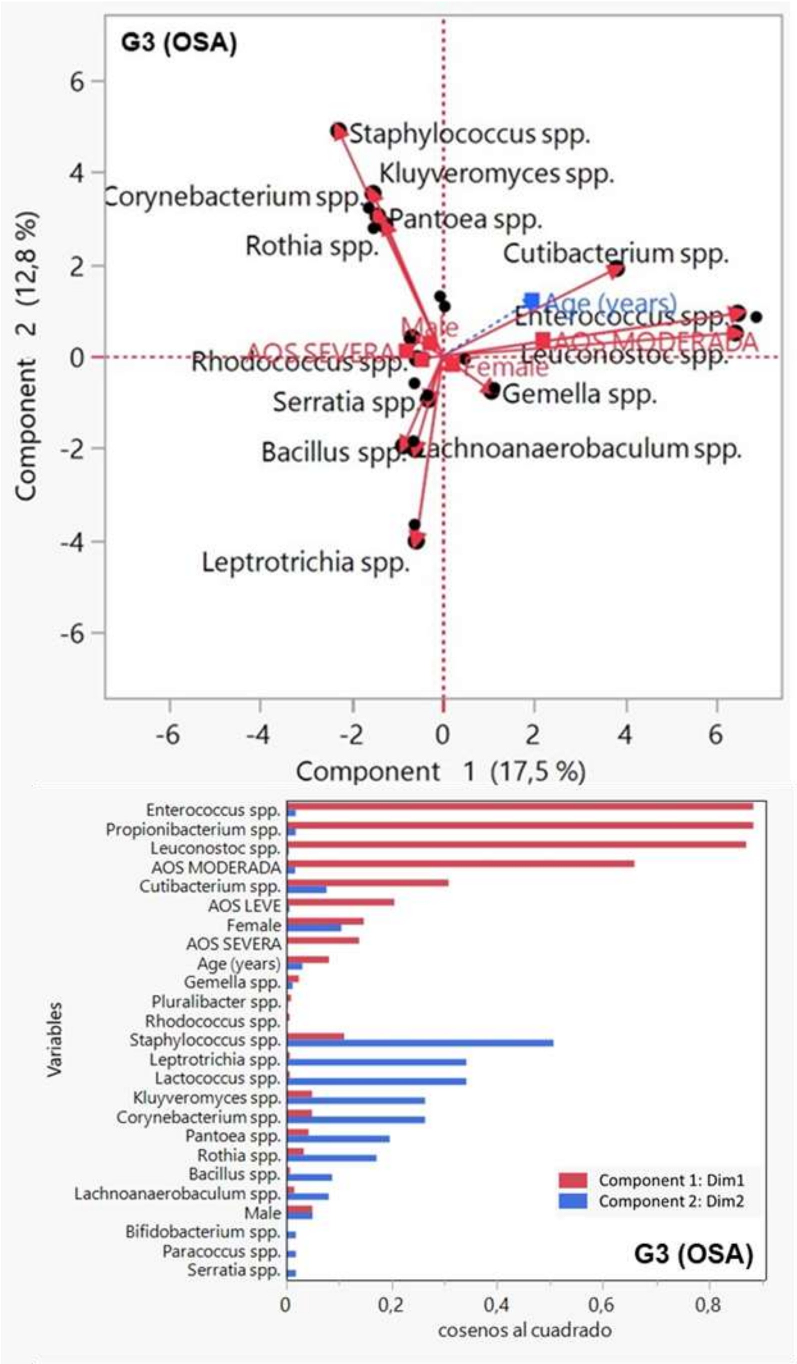

Figure S3. Principal Coordinates Analysis (PCoA) calculated by the relative abundance of microorganisms in G3 (OSA).

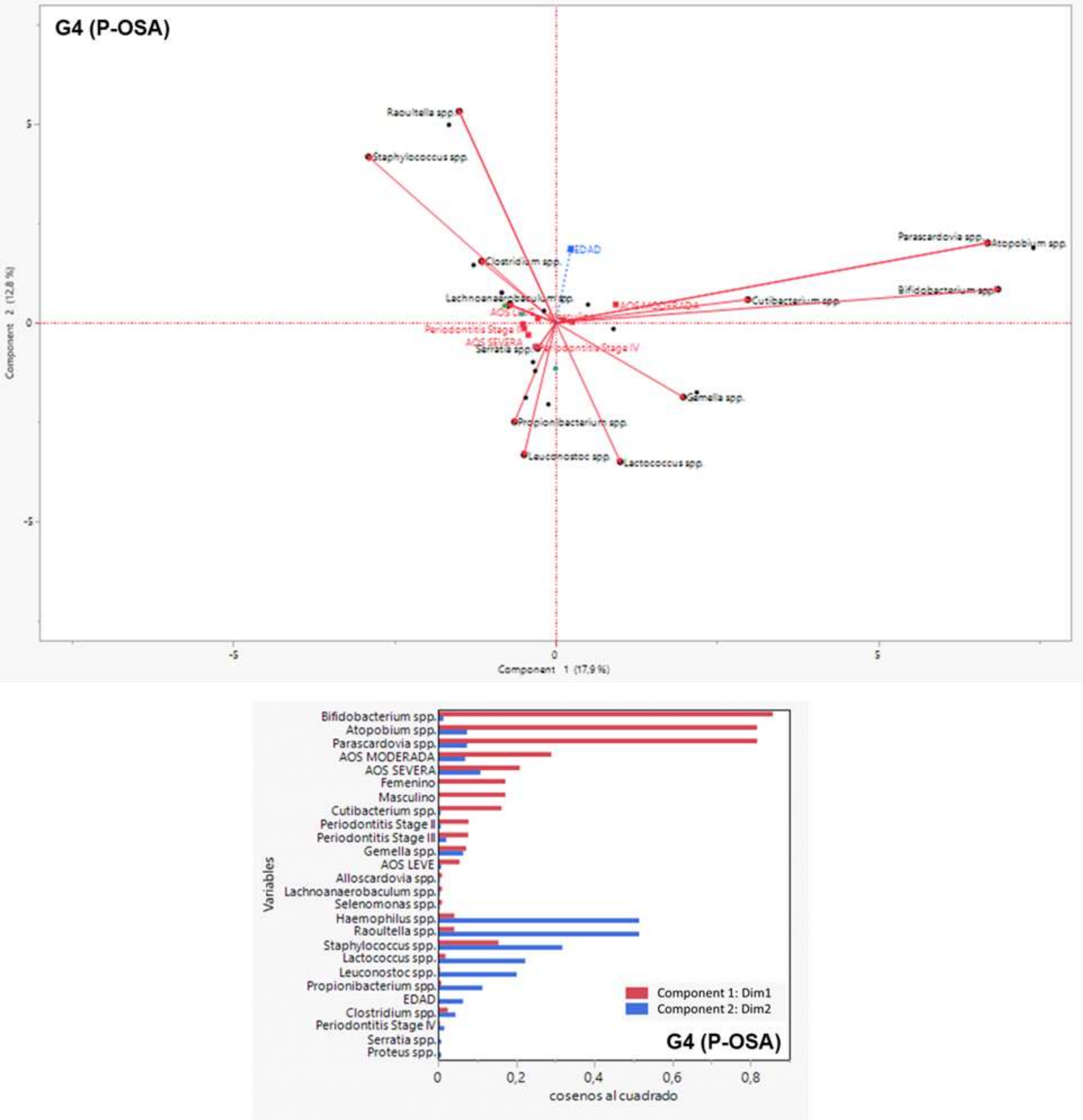

Figure S4. Principal Coordinates Analysis (PCoA) calculated by the relative abundance of microorganisms in G4 (P-OSA).

Table S1.
Species identified in each of group of patients

Species	G1 (H) (n=20)	G2 (P) (n=13)	G3 (OSA) (n=18)	G4 (P-OSA) (n=24)
	Percentage (%)			
Alloscardovia omnicolens	0	3.33	0	4.26
Atopobium parvulum	0	0	0	2.13
Atopobium rimae	0	3.33	0	0
Bacillus weihenstephanensis	0	0	2.50	0
Bifidobacterium dentium	1.89	0	2.50	8.51
Clostridium histolyticum	0	3.33	0	0
Clostridium tertium	0	0	0	2.13
Corynebacterium durum	0	0	2.50	0
Corynebacterium striatum	1.89	0	0	0
Cutibacterium acnes	16.98	13.33	10.0	2.13
Cutibacterium avidum	0	0	0	2.13
Cutibacterium granulosum	0	3.33	0	8.51
Enterococcus faecalis	3.77	3.33	2.50	0
Enterococcus gilvus	1.89	0	0	0
Gemella morbillorum	3.77	0	2.50	2.13
Gemella sanguinis	5.66	0	0	0
Haemophilus parainfluenzae	0	0	0	2.13
Kluyveromyces lactis	0	0	2.50	0
Lachnoanaerobaculum saburreum	1.89	0	5.00	2.13
Lachnoanaerobaculum umeaense	5.66	0	0	0
Lactococcus garvieae	1.89	0	0	4.26
Lactococcus lactis	0	0	0	2.13
Lactococcus raffinolactis	0	0	2.50	2.13
Leptotrichia buccalis	0	0	2.50	0
Leuconostoc citreum	0	0	2.50	0
Leuconostoc lactis	1.89	0	0	2.13
Leuconostoc mesenteroides	0	0	5.00	4.26
Megasphaera micronuciformi	0	3.33	0	0
Neisseria elongata	0	6.67	0	0
Neisseria oralis	0	3.33	0	0
Olsenella uli	0	3.33	0	0
Pantoea agglomerans	0	0	2.50	0
Paracoccus denitrificans	0	0	2.50	0
Parascardovia denticolens	0	3.33	0	2.13
Pluralibacter gergoviae	0	0	2.50	0
Propionibacterium acnes	1.89	0	0	2.13
Propionibacterium freudenreichii	0	0	2.50	0

Propionibacterium propionicum	0	0	0	4.26
Proteus mirabilis	0	0	0	2.13
Pseudopropionibacterium propionicum	0	3.33	0	0
Raoultella ornithinolytica	0	0	0	2.13
Rhodococcus baikonurensis	0	3.33	0	0
Rhodococcus erythropolis	0	3.33	2.50	0
Rothia aeria	0	0	2.50	0
Rothia dentocariosa	5.66	0	0	0
Rothia mucilaginosa	1.89	0	5.00	0
Selenomonas flueggei	0	0	0	2.13
Serratia liquefaciens	0	0	2.50	2.13
Staphylococcus argenteus	0	0	5.00	0
Staphylococcus aureus	11.32	6.67	5.00	6.38
Staphylococcus capitis	0	0	2.50	0
Staphylococcus epidermidis	22.64	23.33	17.50	19.15
Staphylococcus hominis	1.89	10.00	0	2.13
Staphylococcus lugdunensis	1.89	0	0	0
Staphylococcus saccharolyticus	1.89	0	2.50	0
Staphylococcus warneri	3.77	0	2.50	4.26

Table S2.

Positive and negative correlations between the genera of microorganisms and periodontal parameters in each group of patients

Group of patients	Positive correlation with periodontal parameters	Negative correlation with periodontal parameters
**G1**	*Rothia* spp. vs. Missing teeth (*p*=0.001)<br>*Lachnoanaerobaculum* spp. vs. PD (*p*=0.047)<br>*Enterococcus* spp. vs. Sites (%) PD (*p*=0.006)<br>*Leuconostoc* spp. vs. BOP% (*p*=0.048)<br>*Staphylococcus* spp. vs. PI (*p*=0.032)	*Cutibacterium* spp. vs. Missing teeth (*p*=0.005)<br>*Gemella* spp. vs. CAL (*p*=0.032)
**G2**	*Staphylococcus* spp. vs. BOP % (*p*=0.026)	
**G3**	*Lachnoanaerobaculum* spp. vs. CAL (*p*>0.05)<br>*Bacillus* spp. vs. CAL (*p*=0.051)<br>*Bifidobacterium* spp. vs. BOP% (*p*=0.051)<br>*Pluralibacter* spp. vs. Teeth with periodontitis (*p*=0.022)<br>*Rothia* spp. vs. PD (*p*=0.011)<br>*Cutibacterium* spp. vs. Sites (%) PD (*p*=0.030)	*Leuconostoc* spp. vs. PD (*p*=0.046)<br>*Leuconostoc* spp. vs. PI (*p*=0.023)
**G4**	*Proteus* spp. vs. Missing teeth (*p*=0.048)<br>*Proteus* spp. vs. Sites (%) PD (*p*=0.048)<br>*Proteus* spp. vs. CAL (*p*=0.048)<br>*Serratia* spp. vs. Teeth with periodontitis (*p*=0.048)<br>*Serratia* spp. vs. BOP% (*p*=0.048)<br>*Serratia* spp. vs. PI (*p*=0.048)	*Lactococcus* spp. vs. Missing teeth (*p*=0.008)<br>*Propionibacterium* spp. vs. Teeth with periodontitis (*p*=0.003)<br>*Propionibacterium* spp. vs. PD (*p*=0.003)<br>*Propionibacterium* spp. vs. Sites % PD (*p*=0.003)<br>*Propionibacterium* spp. vs. CAL (*p*=0.003)<br>*Propionibacterium* spp. vs. BOP% (*p*=0.003)<br>*Propionibacterium* spp. vs. PI (*p*=0.003)

Table S3.
Identification of cryptic microorganism species and their relation to disease

Microorganism	*Gram*	*Cellular respiration*	*Metabolism*	*Virulence factors*	*Disease*	*Association with systemic diseases*	*References*
Alloscardovia omnicolens	*Positive*	*Anaerobe*	*Fermentative*	*Catalase- and oxidase-negative, nonmotile, non-spore-forming.*	*Dental caries*		*(Ogawa et al., 2016)*
Atopobium parvulum	*Positive*	*Anaerobe*	*Fermentative*		*Halitosis, oral infections, colorectal cancer*		*(Yachida et al., 2019)*
Atopobium rimae	*Positive*	*Anaerobe*	*Fermentative*		*Periodontal abscess, bacteremia.*		*(Chen et al., 2019)*
Bacillus weihenstephanensis	*Positive*	*Anaerobe*	*No fermentative*	*Enterotoxins Hbl and Nhe, sporulate.*			*(Stenfors et al., 2002)*
Bifidobacterium dentium	*Positive*	*Anaerobe*	*Fermentative*	*Adhesins, exoenzymes, protease- and cytokine-modulating molecules, hemolysins.*	*Dental caries*		*(Ventura et al., 2009)*
Clostridium histolyticum	*Positive*	*Facultative Aerobe*	*No fermentative*	*Sporulate, toxins alpha and beta, collagenase, motility-peritrichous flagella*	*Gas gangrene*		*(Sárvári & Schoblocher, 2020)*
Clostridium tertium	*Positive*	*Anaerobe*	*No fermentative*	*Sporulate, toxins*	*Bacteremia, meningitis, septic arthritis, enterocolitis, spontaneous bacterial*		*(Milano et al., 2019)*

					peritonitis, post-traumatic brain abscess, and pneumonia		
Corynebacterium durum	*Positive*	*Aerobic*	*Fermentative*		*Throat, respiratory specimens, blood cultures*		*(Qudeimat et al., 2021)*
Corynebacterium striatum	*Positive*	*facultative anaerobic*	*No fermentative*	*Multidrug resistance.*	*Bacteremia, pneumonia, bronchitis, endocarditis, osteomyelitis, necrotic fasciitis, abscess, wound (MDR)*		*(Alibi et al., 2017)*
Cutibacterium acnes	*Positive*	*Aerobe facultative*	*Fermentative*	*Neuraminidase, lipase, polyunsaturated fatty acid isomerase, heat shock proteins (HSP20, DnaK, DnaJ, GrpE and GroEL), hemolysins and dermatan sulphate-binding adhesins (DsA1 and DsA2).*	*Chronic blepharitis and endophthalmitis.*	*Endocarditis of prosthetic, and native aortic valves.*	*(Singh et al., 2021)*
Cutibacterium avidum	*Positive*	*Aerobe facultative*	*Fermentative*	*Biofilm formation.*	*Bone infections, infective endocarditis (IE), breast infections, abdominal infections, prostate infections, and splenic and skin abscesses*		*(Corvec, 2018)*

Cutibacterium granulosum	*Positive*	*Anaerobic*	*No fermentative*		*Endocarditis, postoperative shoulder infections, and neurosurgical shunt infections*		*(Broly et al., 2020)*
Enterococcus faecalis	*Positive*	*Facultative anaerobe*	*Fermentative*	*Surface adhesins, sex pheromones, lipoteichoic acid, extracellular superoxide production, the lytic enzymes gelatinase and hyaluronidase, and the toxin cytolysin, biofilm.*	*Endodontic disease, bacteremia, endocarditis, urinary tract infections, diabetic foot ulcers, burns, and surgical sites*	*Anterior mitral valve aneurysm as a complication of infective endocarditis. Associated with aerobic vaginitis, and this in turn with preterm labor*	*(Chong et al., 2017)*
Enterococcus gilvus	*Positive*	*Microaeroph ile*	*Fermentative*		*Cholecystitis*		*(Tyrrell et al., 2002)*
Gemella morbillorum	*Positive*	*Capnophilic or microaeroph ilic*	*Fermentative*	*Biofilm formation, capsule, toxins, adhesins, proteases, metal Cofactor Uptake, Phosphorylcholine and Choline-Binding Proteins in the Cell Walls.*	*Septic arthritis and oral abscesses, leading to serious endovascular infections such as endocarditis and pericarditis*	*Diabetes mellitus, hepatorenal dysfunction.*	*(Taimur et al., 2020)*
Gemella sanguinis	*Positive*	*Facultative anaerobic*	*Fermentative*	*Biofilm formation, capsule, toxins, adhesins, proteases, metal Cofactor Uptake, Phosphorylcholine and Choline-Binding*	*Infective endocarditis, bacteremia.*		*(Maraki et al., 2019)*

				Proteins in the Cell Walls.			
Haemophilus parainfluenzae	*Negative*	*Facultative anaerobic*	*Fermentative*	*Capsule, multidrug-resistant, adhesion proteins, pili, the IgA1 protease and, lipooligosaccharide.*	*Endocarditis, meningitis, and pneumonia, otitis media, sinusitis and epiglottitis*		*(Finch et al., 2021)*
Kluyveromyces lactis	*Yeast*	*Aerobic*	*Fermentative*	*pGKL1 killer toxin.*			*(Spohner et al., 2016)*
Lachnoanaerobaculum saburreum	*Positive*	*Anaerobe*	*Fermentative*	*Biofilms formation*	*Endodontic infections.*		*(Grenier, 2013)*
Lactococcus garvieae	*Positive*	*Aerobic*			*Bacteraemia in immune response*		*(Meyburgh et al., 2017)*
Leptotrichia sp	*Negative*	*Facultative anaerobe*	*Saccharolytic*	*Lipopolysaccharide (LPS, endotoxin)*	*Periodontal diseases and abscesses of the oral cavity, endocarditis, severe infections have been reported in immunocompromised patients, septicemia.*	*Diabetes mellitus.*	*(Eribe & Olsen, 2017)*
Leuconostoc citreum	*Positive*	*Facultative anaerobe*	*Fermentative*		*Associated with bacteremia*		*(Modaweb et al., 2022)*
Leuconostoc lactis	*Positive*	*Facultative anaerobe*	*Fermentative*		*Bacterial meningitis, sepsis*		*(Omori et al., 2020)*

Leuconostoc mesenteroides	*Positive*	*Facultative anaerobe*			*Bacteremia*		*(Menegueti et al., 2018)*
Megasphaera micronuciformis	*Negative*	*Obligate anaerobic*	*No fermentative*	*Lipopolysaccharide*	*Periodontal pathogen*	*M. micronuciformis has been isolated from women suffering from preterm birth*	*(Sato et al., 2020)*
Neisseria elongata	*Negative*	*Facultative anaerobe*	*No fermentative*	*Type IV pili*	*Endocarditis and osteomyelitis*		*(Spielman et al., 2020)*
Neisseria oralis	*Negative*	*Facultative anaerobe*	*Fermentative*	*Biofilms formation*		*Only one case of N. oralis as a cause of systemic infection, cystitis in a diabetic adult.*	*(Baniulyte et al., 2021)*
Olsenella uli	*Positive*	*Obligate anaerobic*	*Fermentative*		*Subgingival biofilm in periodontitis*		*(Vieira Colombo et al., 2015)*
Pantoea agglomerans	*Negative*	*Anaerobe*	*Fermentative*	*Lipopolysaccharide (LPS, endotoxin)*	*Abscess, bacteremia, pneumonia, urinary tract infection, septic arthritis, osteomyelitis, peritonitis, choledocholithiasis, dacryocystitis, and endophthalmitis.*		*(Büyükcam et al., 2018)*
Paracoccus denitrificans	*Negative*	*Anaerobe*		*Biofilm formation*			*(Morinaga et al., 2020)*

Parascardovia denticolens	*Positive*	*Anaerobe*	*Fermentative*	*Biofilm formation with S. mutans*	*Dental caries and plaque and peri-implantitis.*		*(Oshima et al., 2015)*
Pluralibacter gergoviae	*Negative*	*Facultative anaerobic*			*Primary bacteraemia, traumatic endophthalmitis, neonatal sepsis, abdominal abscess, pneumonia, osteomyelitis and UTI.*		*(Freire et al., 2016)*
Propionibacterium acnes	*Positive*	*Facultative anaerobic*	*Fermentative*	*Biofilm formation, lipases/esterases, hyaluronate lyase.*	*Acne, endocarditis, spinal Osteomyelitis, endophthalmitis, endodontic infections, periodontitis, prostatitits, meningitis.*	*Participates in the initiation and development of Alzheimer's disease (AD) or Parkinson's disease (PD). Has been associated with aortic aneurysms*	*(McDowell et al., 2021)*
Propionibacterium freudenreichii	*Positive*	*Anaerobe*	*Fermentative*				*(Piwowarek et al., 2018)*
Propionibacterium propionicum	*Positive*	*Anaerobe*	*Fermentative*	*Bacteriocins*	*Endocarditis*		*. (Piwowarek et al., 2018)*

Proteus mirabilis	*Negative*	*Facultative anaerobic*	*Fermentative*	*Adhesion proteins, quorum sensing molecules, lipopolysaccharides, efflux pumps, and urease enzyme*	*Catheter-associated urinary tract infections (CAUTIs)*		*(Armbruster et al., 2018)*
Pseudopropionibacterium propionicum	*Positive*	*Anaerobe*	*Fermentative*		*Actinomycosis. Radicular and extra-radicular infections. Lacrimal canaliculitis, cervicofacial infections, tympanomastoiditis, pulmonary infections, osteomyelitis.*		*(Suzuki et al., 2019)*
Raoultella ornithinolytica	*Negative*	*Facultative anaerobe*		*Lipopolysaccharide, polysaccharide capsule, fimbriae, siderophores, toxin, hydrolytic enzymes, bacteriocins and biofilm*	*Dentin of infected root canals and causes urinary tract infections, gastrointestinal, hepatobiliary, osteoarticular*	*Associated with infection in the foot of a diabetic patient*	*(Sękowska, 2017)*

Rhodococcus erythropolis	*Positive*	*Obligate aerobic*	*Fermentative*	*Capsule*	*Bloodstream infection, peritonitis, disseminated skin infection, chronic endophthalmitis, meningoencephalitis.*		*(Baba et al., 2009)*
Rothia aeria	*Positive*	*Facultative anaerobe*	*Fermentative*	*Biofilm formation*	*Bacteremia, septic arthritis, ovarian tube abscess, cervical abscesses, infections of the respiratory tract and endocarditis.*		*(Greve et al., 2021)*
Rothia dentocariosa	*Positive*	*Facultative anaerobe*	*Fermentative*	*Polysaccharide RPS*	*Cerebral empyema, bone and joint infections, endophthalmitis, skin abscess, pyelonephritis, infected ascites, peritoneal dialysis-related peritonitis, catheter-associated infections, and bacteremia.*		*(Franconieri et al., 2020)*

Rothia mucilaginosa	*Positive*	*Aerobic*		*Enterobactin production. Encapsulated*		*Cases of bacteremia, endocarditis, catheter-associated bloodstream infection, central nervous system infections, endophthalmitis,osteomyelitis, prosthetic joint infection, pneumonia, cholangitis.*	*(Uranga et al., 2020)*
Selenomonas flueggei	*Negative*	*Obligate anaerobic*	*Fermentative*	*The lipopolysaccharide purified from these bacterial species has been shown to possess several endotoxic properties in mice*	*Play a role in the pathogenesis of periodontal disease.*		*(Enigk et al., 2020)*
Serratia liquefaciens	*Negative*	*Facultative anaerobe*	*Fermentative*	*DNase, lipase, gelatinase, hemolysin, proteases, chitinase, chloroperoxidase, and multiple isozymes of alkaline phosphatase, biofilm formation.*	*Peritonitis, fistulous pyoderma, septic arthritis, ocular infections.*		*(Begrem et al., 2021)*

Staphylococcus argenteus	*Positive*	*Aerobic*	*Fermentative*	*Coagulase-positive, enterotoxins, TSST-1, exfoliative toxins, Panton–Valentine leucocidin (PVL), adhesins.*	*Skin and soft tissue infections.*		*(Jiang et al., 2018)*
Staphylococcus aureus	*Positive*	*Anaerobic facultative*	*Fermentative*	*Coagulase-positive, enterotoxins, TSST-1, exfoliative toxins, Panton–Valentine leucocidin (PVL), adhesins.*	*Bloodstream infections, Endocarditis,Osteomyelitis, Lung infection, suppurative diseases, food poisoning, pneumonia, and toxic shock syndrome, prosthetic joint infections.*		*(Ahmad-Mansour et al., 2021)*
Staphylococcus capitis	*Positive*	*Aerobic*	*Fermentative*	*Enterotoxins, TSST-1, exfoliative toxins, Panton–Polysaccharide intercellular adhesin (PIA), adhesins, biofilm formation.*	*Endocarditis, urinary tract infection.*		*(Cui et al., 2013)*
Staphylococcus epidermidis	*Positive*	*Aerobic*	*Fermentative*	*Adhesins such as autolysin (AtlE) , extracellular DNA (eDNA), SSP-1, SSP-2, biofilm formation, antibiotic resistance.*	*Skin diseases, such as atopic dermatitis or psoriasis.*		*(Brescó et al., 2017)*

Staphylococcus hominis	*Positive*	*Aerobic*	*Fermentative*	*Adhesins :surface-binding proteins and exopolymers, extracellular proteases, metalloproteases, lipases and esterases.*	*Bloodstream infections, endocarditis, peritonitis, osteomyelitis, bone and joint infections.*	*(Szczuka et al., 2018)*
Staphylococcus lugdunensis	*Positive*	*Aerobic*	*No fermentative*	*Coagulase negative-adhesins :surface-binding proteins and exopolymers, extracellular proteases, metalloproteases, lipases and esterases*	*Central nervous system infections, endocarditis, endophthalmitis, osteomyelitis, peritonitis, prosthetic joint infections, urinary tract infections, and systemic infections*	*(Parthasarathy et al., 2020)*
Staphylococcus saccharolyticus	*Positive*	*Anaerobic*	*Saccharolytic*	*Biofilm formation, protective exopolymers such as PNAG and PGA*	*Endocarditis, spondylodiscitis, bone marrow infections, pneumonia, and pyomyositis*	*(Wang et al., 2020)*
Staphylococcus warneri	*Positive*	*Positive*	*Facultative anaerobe*	*Fermentative*	*Proteases, peptidoglycan hydrolases, hemolysins, enterotoxins, coagulase, lipases*	*(Kanuparthy et al., 2020)*

References:

1. Ahmad-Mansour, N., Loubet, P., Pouget, C., Dunyach-Remy, C., Sotto, A., Lavigne, J. P., & Molle, V. (2021). Staphylococcus aureus toxins: An update on their pathogenic properties and potential treatments. *Toxins*, *13*(10), 1–22. https://doi.org/10.3390/toxins13100677
2. Alibi, S., Ferjani, A., Boukadida, J., Cano, M. E., Fernández-Martínez, M., Martínez-Martínez, L., & Navas, J. (2017). Occurrence of Corynebacterium striatum as an emerging antibiotic-resistant nosocomial pathogen in a Tunisian hospital. *Scientific Reports*, *7*(1), 1–8. https://doi.org/10.1038/s41598-017-10081-y
3. Armbruster, C. E., Mobley, H. L. T., Pearson, M. M., Arbor, A., States, U., & States, U. (2018). *Pathogenesis of Proteus mirabilis Infection*. *8*(1), 1–123. https://doi.org/10.1128/ecosalplus.ESP-0009-2017.Pathogenesis
4. Baba, H., Nada, T., Ohkusu, K., Ezaki, T., Hasegawa, Y., & Paterson, D. L. (2009). First case of bloodstream infection caused by Rhodococcus erythropolis. *Journal of Clinical Microbiology*, *47*(8), 2667–2669. https://doi.org/10.1128/JCM.00294-09
5. Baniulyte, G., Svirpliene, S., Eccleston, A., Arjunan, S., & Connor, M. (2021). Neisseria oralis septicaemia in a newborn: first recorded case. *Paediatrics and International Child Health*, *41*(3), 226–227. https://doi.org/10.1080/20469047.2020.1826780
6. Begrem, S., Jérôme, M., Leroi, F., Delbarre-Ladrat, C., Grovel, O., & Passerini, D. (2021). Genomic diversity of Serratia proteamaculans and Serratia liquefaciens predominant in seafood products and spoilage potential analyses. *International Journal of Food Microbiology*, *354*(July). https://doi.org/10.1016/j.ijfoodmicro.2021.109326
7. Brescó, M. S., Harris, L. G., Thompson, K., Stanic, B., Morgenstern, M., O'Mahony, L., Richards, R. G., & Moriarty, T. F. (2017).

Pathogenic mechanisms and host interactions in Staphylococcus epidermidis device-related infection. *Frontiers in Microbiology*, *8*(AUG). https://doi.org/10.3389/fmicb.2017.01401

8. Broly, M., Ruffier d'Epenoux, L., Guillouzouic, A., Le Gargasson, G., Juvin, M. E., Leroy, A. G., Bémer, P., & Corvec, S. (2020). Propionibacterium/Cutibacterium species–related positive samples, identification, clinical and resistance features: a 10-year survey in a French hospital. *European Journal of Clinical Microbiology and Infectious Diseases*, *39*(7), 1357–1364. https://doi.org/10.1007/s10096-020-03852-5
9. Büyükcam, A., Tuncer, Ö., Gür, D., Sancak, B., Ceyhan, M., Cengiz, A. B., & Kara, A. (2018). Clinical and microbiological characteristics of Pantoea agglomerans infection in children. *Journal of Infection and Public Health*, *11*(3), 304–309. https://doi.org/10.1016/j.jiph.2017.07.020
10. Chen, J., Wu, X., Zhu, D., Xu, M., Yu, Y., Yu, L., & Zhang, W. (2019). Microbiota in Human Periodontal Abscess Revealed by 16S rDNA Sequencing. *Frontiers in Microbiology*, *10*(July), 1–12. https://doi.org/10.3389/fmicb.2019.01723
11. Chong, K. K. L., Tay, W. H., Janela, B., Yong, A. M. H., Liew, T. H., Madden, L., Keogh, D., Barkham, T. M. S., Ginhoux, F., Becker, D. L., & Kline, K. A. (2017). Enterococcus faecalis Modulates Immune Activation and Slows Healing during Wound Infection. *Journal of Infectious Diseases*, *216*(12), 1644–1654. https://doi.org/10.1093/infdis/jix541
12. Corvec, S. (2018). Clinical and biological features of Cutibacterium (Formerly Propionibacterium) avidum, an underrecognized microorganism. *Clinical Microbiology Reviews*, *31*(3), 1–42. https://doi.org/10.1128/CMR.00064-17
13. Cui, B., Smooker, P. M., Rouch, D. A., Daley, A. J., & Deighton, M. A. (2013). Differences between two clinical Staphylococcus capitis subspecies as revealed by biofilm, antibiotic resistance, and pulsed-field gel electrophoresis profiling. *Journal of Clinical Microbiology*, *51*(1), 9–14. https://doi.org/10.1128/JCM.05124-11
14. Enigk, K., Jentsch, H., Rodloff, A. C., Eschrich, K., & Stingu, C. S. (2020). Activity of five antimicrobial peptides against periodontal as well as non-periodontal pathogenic strains. *Journal of Oral Microbiology*, *12*(1). https://doi.org/10.1080/20002297.2020.1829405
15. Eribe, E. R. K., & Olsen, I. (2017). Leptotrichia species in human infections II. *Journal of Oral Microbiology*, *9*(1). https://doi.org/10.1080/20002297.2017.1368848
16. Finch, L. C., Gerdzhikov, S., & Buttery, R. (2021). Haemophilus parainfluenzae endocarditis presenting with symptoms of COVID-19. *BMJ Case Reports*, *14*(8), 4–9. https://doi.org/10.1136/bcr-2021-245210
17. Franconieri, F., Join-Lambert, O., Creveuil, C., Auzou, M., Labombarda, F., Aouba, A., Verdon, R., & de La Blanchardière, A. (2020). Rothia spp. infective endocarditis: A systematic literature review. *Medecine et Maladies Infectieuses*, *51*, 228–235. https://doi.org/10.1016/j.medmal.2020.10.021
18. Freire, M. P., De Oliveira Garcia, D., Cury, A. P., Spadão, F., Di Gioia, T. S. R., Francisco, G. R., Bueno, M. F. C., Tomaz, M., De

Paula, F. J., De Faro, L. B., Piovesan, A. C., Rossi, F., Levin, A. S., David Neto, E., Nahas, W. C., & Pierrotti, L. C. (2016). Outbreak of IMP-producing carbapenem-resistant Enterobacter gergoviae among kidney transplant recipients. *Journal of Antimicrobial Chemotherapy*, *71*(9), 2577–2585. https://doi.org/10.1093/jac/dkw165

19. Grenier, D. (2013). Porphyromonas gingivalis outer membrane vesicles mediate coaggregation and piggybacking of Treponema denticola and Lachnoanaerobaculum saburreum. *International Journal of Dentistry*, *2013*. https://doi.org/10.1155/2013/305476
20. Greve, D., Moter, A., Kleinschmidt, M. C., Pfäfflin, F., Stegemann, M. S., Kursawe, L., Grubitzsch, H., Falk, V., & Kikhney, J. (2021). Rothia aeria and Rothia dentocariosa as biofilm builders in infective endocarditis. *International Journal of Medical Microbiology*, *311*(2). https://doi.org/10.1016/j.ijmm.2021.151478
21. Hedberg, M. E., Moore, E. R. B., Svensson-Stadler, L., Hörstedt, P., Baranov, V., Hernell, O., Wai, S. N., Hammarström, S., & Hammarström, M. L. (2012). Lachnoanaerobaculum gen. nov., a new genus in the Lachnospiraceae: Characterization of Lachnoanaerobaculum umeaense gen. nov., sp. nov., isolated from the human small intestine, and Lachnoanaerobaculum orale sp. nov., isolated from saliva, and reclassification of Eubacterium saburreum (Prévot 1966) Holdeman and Moore 1970 as Lachnoanaerobaculum saburreum comb. nov. *International Journal of Systematic and Evolutionary Microbiology*, *62*(11), 2685–2690. https://doi.org/10.1099/ijs.0.033613-0
22. Jiang, B., You, B., Tan, L., Yu, S., Li, H., Bai, G., Li, S., Rao, X., Xie, Z., Shi, X., Peng, Y., & Hu, X. (2018). Clinical Staphylococcus argenteus develops to small colony variants to promote persistent infection. *Frontiers in Microbiology*, *9*(JUN), 1–10. https://doi.org/10.3389/fmicb.2018.01347
23. Jung, M. Y., Lee, C., Seo, M. J., Roh, S. W., & Lee, S. H. (2020). Characterization of a potential probiotic bacterium Lactococcus raffinolactis WiKim0068 isolated from fermented vegetable using genomic and in vitro analyses. *BMC Microbiology*, *20*(1), 1–10. https://doi.org/10.1186/s12866-020-01820-9
24. Kanuparthy, A., Challa, T., Meegada, S., Siddamreddy, S., & Muppidi, V. (2020). Staphylococcus warneri: Skin Commensal and a Rare Cause of Urinary Tract Infection. *Cureus*, *12*(5). https://doi.org/10.7759/cureus.8337
25. Kleerebezem, M., Bachmann, H., van Pelt-KleinJan, E., Douwenga, S., Smid, E. J., Teusink, B., & van Mastrigt, O. (2020). Lifestyle, metabolism and environmental adaptation in Lactococcus lactis. *FEMS Microbiology Reviews*, *44*(6), 804–820. https://doi.org/10.1093/femsre/fuaa033
26. Li, Y., Kawamura, Y., Fujiwara, N., Naka, T., Lui, H., Huang, X., Kobayashi, K., & Ezaki, T. (2004). Rothia aeria sp. nov., Rhodococcus baikonurensis sp. nov. and Arthrobacter russicus sp. nov., isolated from air in the Russian space laboratory Mir. *International Journal of Systematic and Evolutionary Microbiology*, *54*(3), 827–835. https://doi.org/10.1099/ijs.0.02828-0
27. Maraki, S., Plevritaki, A., Kofteridis, D., Scoulica, E., Eskitzis, A., Gikas, A., & Panagiotakis, S. H. (2019). Bicuspid aortic valve

endocarditis caused by Gemella sanguinis: Case report and literature review. *Journal of Infection and Public Health*, *12*(3), 304–308. https://doi.org/10.1016/j.jiph.2019.01.001
28. McDowell, A., McLaughlin, J., & Layton, A. M. (2021). Is Cutibacterium (previously Propionibacterium) acnes a potential pathogenic factor in the aetiology of the skin disease progressive macular hypomelanosis? *Journal of the European Academy of Dermatology and Venereology*, *35*(2), 338–344. https://doi.org/10.1111/jdv.16789
29. Menegueti, M. G., Gaspar, G. G., Laus, A. M., Basile-Filho, A., Bellissimo-Rodrigues, F., & Auxiliadora-Martins, M. (2018). Bacteremia by Leuconostoc mesenteroides in an immunocompetent patient with chronic Chagas disease: A case report. *BMC Infectious Diseases*, *18*(1), 13–15. https://doi.org/10.1186/s12879-018-3452-7
30. Meyburgh, C. M., Bragg, R. R., & Boucher, C. E. (2017). Lactococcus garvieae: An emerging bacterial pathogen of fish. *Diseases of Aquatic Organisms*, *123*(1), 67–79. https://doi.org/10.3354/dao03083
31. Milano, V., Biehle, L., Patel, S., & Hammer, J. (2019). Clostridium tertium bacteremia and hepatic abscess in a non-neutropenic patient. *IDCases*, *15*, e00510. https://doi.org/10.1016/j.idcr.2019.e00510
32. Modaweb, A., Mansoor, Z., Alsarhan, A., & Abuhammour, W. (2022). A Case of Successfully Treated Central Line-Associated Bloodstream Infection Due to Vancomycin-Resistant Leuconostoc Citreum in a Child With Biliary Atresia. *Cureus*, *14*(1), 12–15. https://doi.org/10.7759/cureus.21227
33. Morinaga, K., Yoshida, K., Takahashi, K., Nomura, N., & Toyofuku, M. (2020). Peculiarities of biofilm formation by Paracoccus denitrificans. *Applied Microbiology and Biotechnology*, *104*(6), 2427–2433. https://doi.org/10.1007/s00253-020-10400-w
34. Ogawa, Y., Koizumi, A., Kasahara, K., Lee, S. T., Yamada, Y., Nakano, R., Yano, H., & Mikasa, K. (2016). Bacteremia secondary to Alloscardovia omnicolens urinary tract infection. *Journal of Infection and Chemotherapy*, *22*(6), 424–425. https://doi.org/10.1016/j.jiac.2015.12.013
35. Omori, R., Fujiwara, S., Ishiyama, H., Kuroda, H., & Kohara, N. (2020). Leuconostoc lactis- A Rare Cause of Bacterial Meningitis in an Immunocompromised Host. *Internal Medicine*, *59*(22), 2935–2936. https://doi.org/10.2169/internalmedicine.5076-20
36. Oshima, K., Hayashi, J. I., Toh, H., Nakano, A., Omori, E., Hattori, Y., Morita, H., Honda, K., & Hattori, M. (2015). Complete genome sequence of Scardovia inopinata JCM 12537T, isolated from human dental caries. *Genome Announcements*, *3*(3), 5–6. https://doi.org/10.1128/genomeA.00481-15
37. Parthasarathy, S., Shah, S., Raja Sager, A., Rangan, A., & Durugu, S. (2020). Staphylococcus lugdunensis: Review of Epidemiology, Complications, and Treatment. *Cureus*, *12*(6), 6–13. https://doi.org/10.7759/cureus.8801
38. Piwowarek, K., Lipińska, E., Hać-Szymańczuk, E., Kieliszek, M., & Ścibisz, I. (2018). Propionibacterium spp.—source of propionic acid, vitamin B12, and other metabolites important for the industry. *Applied Microbiology and Biotechnology*, *102*(2), 515–538.

https://doi.org/10.1007/s00253-017-8616-7
39. Qudeimat, M. A., Alyahya, A., Karched, M., Behbehani, J., & Salako, N. O. (2021). Dental plaque microbiota profiles of children with caries-free and caries-active dentition. *Journal of Dentistry*, *104*(December 2019), 103539. https://doi.org/10.1016/j.jdent.2020.103539
40. Sárvári, K. P., & Schoblocher, D. (2020). The antibiotic susceptibility pattern of gas gangrene-forming Clostridium spp. clinical isolates from South-Eastern Hungary. *Infectious Diseases*, *52*(3), 196–201. https://doi.org/10.1080/23744235.2019.1696472
41. Sato, N., Kakuta, M., Hasegawa, T., Yamaguchi, R., Uchino, E., Kobayashi, W., Sawada, K., Tamura, Y., Tokuda, I., Murashita, K., Nakaji, S., Imoto, S., Yanagita, M., & Okuno, Y. (2020). Metagenomic analysis of bacterial species in tongue microbiome of current and never smokers. *Npj Biofilms and Microbiomes*, *6*(1), 1–9. https://doi.org/10.1038/s41522-020-0121-6
42. Sękowska, A. (2017). Raoultella spp.—clinical significance, infections and susceptibility to antibiotics. *Folia Microbiologica*, *62*(3), 221–227. https://doi.org/10.1007/s12223-016-0490-7
43. Singh, M., Teles, F., Uzel, N. G., & Papas, A. (2021). Characterizing Microbiota from Sjögren's Syndrome Patients. *JDR Clinical and Translational Research*, *6*(3), 324–332. https://doi.org/10.1177/2380084420940623
44. Spielman, A. F., Ghumman, A., Panthaki, Z., & Lessard, A. S. (2020). Neisseria elongata osteomyelitis: Literature review and case report in a 63-year-old male presenting with progressive right-handed redness, swelling and pain. *International Journal of Surgery Case Reports*, *73*, 228–230. https://doi.org/10.1016/j.ijscr.2020.07.022
45. Spohner, S. C., Schaum, V., Quitmann, H., & Czermak, P. (2016). Kluyveromyces lactis: An emerging tool in biotechnology. *Journal of Biotechnology*, *222*, 104–116. https://doi.org/10.1016/j.jbiotec.2016.02.023
46. Stenfors, L. P., Mayr, R., Scherer, S., & Granum, P. E. (2002). Pathogenic potential of fifty Bacillus weihenstephanensis strains. *FEMS Microbiology Letters*, *215*(1), 47–51. https://doi.org/10.1016/S0378-1097(02)00891-1
47. Suzuki, H., Arshava, E. V., Ford, B., & Nauseef, W. M. (2019). Don't let its name fool you: Relapsing thoracic actinomycosis caused by pseudopropionibacterium propionicum (formerly propionibacterium propionicum). *American Journal of Case Reports*, *20*, 1961–1965. https://doi.org/10.12659/AJCR.919775
48. Szczuka, E., Krzymińska, S., Bogucka, N., & Kaznowski, A. (2018). Multifactorial mechanisms of the pathogenesis of methicillin-resistant Staphylococcus hominis isolated from bloodstream infections. *Antonie van Leeuwenhoek, International Journal of General and Molecular Microbiology*, *111*(7), 1259–1265. https://doi.org/10.1007/s10482-017-1007-3
49. Taimur, S., Madiha, R., Samar, F., & Bushra, J. (2020). Gemella morbillorum endocarditis in a patient with a bicuspid aortic valve. *Hellenic Journal of Cardiology*, *51*(2), 183–186. https://doi.org/10.14744/nci.2020.39206
50. Tyrrell, G. J., Turnbull, L. A., Teixeira, L. M., Lefebvre, J., Carvalho, M. da G. S., Facklam, R. R., & Lovgren, M. (2002). Enterococcus gilvus sp. nov. and Enterococcus pallens sp. nov. isolated from human clinical specimens. *Journal of Clinical Microbiology*, *40*(4),

1140–1145. https://doi.org/10.1128/JCM.40.4.1140-1145.2002
51. Uranga, C. C., Arroyo, P., Duggan, B. M., Gerwick, W. H., & Edlund, A. (2020). Commensal Oral Rothia mucilaginosa Produces Enterobactin, a Metal-Chelating Siderophore. *MSystems*, *5*(2), 1–14. https://doi.org/10.1128/msystems.00161-20
52. Ventura, M., Turroni, F., Zomer, A., Foroni, E., Giubellini, V., Bottacini, F., Canchaya, C., Claesson, M. J., He, F., Mantzourani, M., Mulas, L., Ferrarini, A., Gao, B., Delledonne, M., Henrissat, B., Coutinho, P., Oggioni, M., Gupta, R. S., Zhang, Z., … Van Sinderen, D. (2009). The bifidobacterium dentium Bd1 genome sequence reflects its genetic adaptation to the human oral cavity. *PLoS Genetics*, *5*(12). https://doi.org/10.1371/journal.pgen.1000785
53. Vieira Colombo, A. P., Magalhães, C. B., Hartenbach, F. A. R. R., Martins do Souto, R., & Maciel da Silva-Boghossian, C. (2015). Periodontal-disease-associated biofilm: A reservoir for pathogens of medical importance. *Microbial Pathogenesis*, *94*, 27–34. https://doi.org/10.1016/j.micpath.2015.09.009
54. Wang, P., Liu, Y., Xu, Y., & Xu, Z. (2020). Staphylococcus saccharolyticus infection: case series with a PRISMA-compliant systemic review. *Medicine*, *99*(26), e20686. https://doi.org/10.1097/MD.0000000000020686
55. Yachida, S., Mizutani, S., Shiroma, H., Shiba, S., Nakajima, T., Sakamoto, T., Watanabe, H., Masuda, K., Nishimoto, Y., Kubo, M., Hosoda, F., Rokutan, H., Matsumoto, M., Takamaru, H., Yamada, M., Matsuda, T., Iwasaki, M., Yamaji, T., Yachida, T., … Yamada, T. (2019). Metagenomic and metabolomic analyses reveal distinct stage-specific phenotypes of the gut microbiota in colorectal cancer. *Nature Medicine*, *25*(6), 968–976. https://doi.org/10.1038/s41591-019-0458-7
